# The role of Advanced Practice Providers in improving primary care capacity: practice perspectives on challenges and opportunities

**DOI:** 10.3389/fmed.2026.1885926

**Published:** 2026-09-03

**Authors:** Marie Shoen, Ben Webel, Jacqueline Britz, Hailee Hoffman, Jennifer Gilbert, Alex H. Krist, E. Marshall Brooks

**Affiliations:** 1 Department of Family Medicine and Population Health, Virginia Commonwealth University, Richmond, VA, United States; 2 School of Nursing, Simmons University, Boston, MA, United States

**Keywords:** health workforce, nurse practitioner, physician assistant, primary care, primary care shortage, retention, rural health, turnover

## Abstract

**Background:**

In Virginia and across the United States, primary care workforce shortages limit access to care and exacerbate adverse health outcomes. We conducted a statewide survey of primary care practices in 2022 and found that 42% of practices reported losing a physician or Advanced Practice Provider (APP), including Nurse Practitioners (NPs) and Physician Assistants (PAs), in the past year. Adding APPs to primary care teams is one strategy practices are using to mitigate the shrinking availability of primary care physicians. However, APPs face workforce challenges that increase their susceptibility to turnover.

**Methods:**

Thirteen semi-structured interviews were conducted with fourteen physicians, managers, and administrators within primary care practices in Virginia Health Professional Shortage Areas (HPSAs) that reported losing a clinician in the past year. Participants were asked about experiences with turnover, impacts of turnover, and retention strategies. The role of APPs emerged as a prominent topic across ten interviews. The ten interviews containing discussions around APPs were transcribed and thematically analyzed for this study.

**Results:**

According to practice representatives, APPs increased primary care capacity at an efficient cost, but practices frequently struggled with APP retention. Participants described numerous reasons for APP departures, including changing specialties, relocating, and seeking higher pay. Turnover was reportedly more common among APPs with limited primary care experience and repeatedly attributed to perceived discomfort with patient complexity or volume. In contrast, some cited a lack of autonomy as a reason for APPs leaving their role. To improve APP retention, participants suggested implementing phased onboarding, intensive mentorship via interprofessional care teams, and ongoing professional development.

**Conclusions:**

APPs are viewed as a solution to the primary care workforce shortage, but turnover is common and negatively impacts practices and patients. Targeted support could help mitigate some of the obstacles faced by APPs to improve retention. This work was funded by the National Institute of Nursing Research (NINR) Award 75N98023F00078.

## 1 Introduction

Primary care access and utilization contribute to positive health outcomes and health experiences across U.S. populations (1–3). Primary care capacity is insufficient in many communities, threatening access to preventative services, chronic care, care coordination, and more (4, 5). Limited access to primary care services is driven by widespread and accelerating primary care physician (PCP) shortages (6, 7). The total number of PCPs across the U.S. increased from 2005 to 2015, but the mean PCP supply decreased by over five PCPs per 100,000 population, primarily due to population increases and disproportionate PCP losses in some areas (6). The Association of American Medical Colleges (AAMC) projects a continued total physician shortage of between 13,500 and 86,000 by 2036, driven primarily by population growth and aging, an aging physician workforce nearing retirement, and the pace of graduate medical education funding growth (7, 8). A separate analysis by the Health Resources and Services Administration (HRSA) similarly projects a shortage of 68,020 primary care physicians by 2036, representing more than 80% of its projected total physician shortfall (9). A recent study in Virginia found that the primary care workforce shortage may be even more dire than prior estimates (10), revealing that only 56% of census tracts had adequate access to PCPs, with rural communities disproportionately impacted (11).

In Virginia and across the U.S., the decline of PCP availability is contrasted with a proliferation of the Advanced Practice Provider (APP) workforce. APPs, including Nurse Practitioners (NPs) and Physician Assistants (PAs), are health professionals who have received specialized graduate-level medical training and are licensed to diagnose, manage, and treat medical conditions. APPs currently make up nearly 40% of U.S. healthcare providers (12) and are often promoted as a key solution to the primary care workforce crisis, particularly as HRSA projects the national NP workforce will reach 176% of demand and the PA workforce 108% of demand by 2037 (13). The American Association of Nurse Practitioners (AANP) 2024 Workforce Survey identified over 460,000 NPs licensed in the U.S., approximately 87% of whom trained in programs focused on primary care (14). As a result, primary care now employs nearly 20,000 more APPs than physicians (9, 15). In addition to their widespread availability and substantial scope of practice, APPs offer a range of benefits to primary care teams and patients, such as improving access to care, care quality, continuity of care, and patient satisfaction (16–19).

Despite their contributions to addressing the primary care shortage, PCPs and APPs face an assortment of professional challenges, like high case volume, inadequate staffing, long hours, and burnout. Unique factors such as pay inequity and inconsistent training, orientation, and autonomy make APPs particularly vulnerable to turnover (20–23). In the primary care setting, clinician turnover may lead to worsened patient experiences and decreased likelihood of receiving timely care (24), contributing to higher emergency department, specialty, and urgent care use (25). These issues are especially acute in areas already facing healthcare workforce shortages. The U.S. Department of Health and Human Services developed Health Professional Shortage Area (HPSA) designations to map and address areas with health care access challenges (26, 27). In Virginia, HPSAs span both urban and rural areas, but shortages are often more pronounced in rural areas due to heightened access and personnel challenges (28, 29). Nationally, HRSA projects primary care supply will meet only 52% of nonmetro demand by 2036, compared to 99% of metro demand (9). The growth of the APP workforce presents a possible solution to clinician shortages in rural parts of the country, but policy and regulatory barriers inhibit independent practice in some areas (28, 30). Existing rural recruitment strategies, such as loan repayment programs, offer short-term hiring benefits, but additional research and support is needed to promote long-term retention (31).

Despite the growing APP workforce and the significant role of APPs within many care teams, APP turnover within and across medical settings is not thoroughly understood (32). Our study aims to characterize APP turnover in primary care practices located in HPSAs across Virginia and examine practice perspectives on contributors to turnover, impacts of turnover, and strategies to reduce turnover. Developing our understanding of APP experiences in diverse primary care settings can support efforts to improve APP job satisfaction and retention. In turn, increased retention and job satisfaction of APPs can reduce the negative impacts of primary care clinician shortages on practices and patients.

## 2 Methods

### 2.1 Study setting

We studied Virginia primary care practices located in HPSAs as defined by the Health Resources and Services Administration (HRSA) that lost one or more physician, NP, or PA in the prior year. The Virginia Ambulatory Care Outcomes Research Network (ACORN) maintains a database of every primary care practice and primary care clinician (MD, DO, NP, and PA) in Virginia with clinicians nested into practices. This includes the specialties of family medicine, general internal medicine, pediatrics, and women's health. Every four years, we survey every primary care practice in Virginia to assess the state of primary care. In 2022, 526 out of 2,296 primary care practices (23%) completed the survey, with broad representation across geography, ownership, and payer mix. We included a question about major changes experienced by practices in the past year and 222/526 (42.2%) practices reported losing at least one doctor, nurse practitioner, or physician assistant in the prior year due to planned retirement, early retirement, moving, change of practices, being fired, or death. Of those who reported losing a clinician in the prior year, 114/222 (51.4%) were located in Geographic, High Needs Geographic, or Low Income Population HPSAs, parts of a state where a defined area is experiencing clinician shortages.

Virginia currently has 56 geographic HPSAs and 44 population HPSAs (26, 27). A Geographic Primary Care HPSA designates a shortage of primary care clinicians for an entire group of people within a defined geographic area (26). A High Needs Geographic Primary Care HPSA is a designated area with a severe, chronic shortage of primary care clinicians relative to its population (26). Federal regulation requires that in order to receive Primary Care HPSA designation, an area's population-to-primary care clinician ratio be at least 3,500 to 1, and 3,000 to 1 if there are high needs in the community (27). A Low-Income Population Primary Care HPSA denotes a shortage of primary care clinicians within a defined geographic area for low-income people specifically (26). Every practice that answered, “Lost one or more doctor, nurse practitioner, or physician assistant” and was located in a Geographic Primary Care HPSA, High Needs Geographic Primary Care HPSA, or a Low-Income Population Primary Care HPSA was invited to participate in an interview.

### 2.2 Data collection and analysis

The development of the semi-structured interview guide was informed by the academic literature on primary care interdisciplinary teamwork, workforce development and turnover, and practice organizational stress. It was structured into three main thematic domains to explore the impact of clinician turnover on team-based care in primary care practices. This included 1) factors contributing to clinician loss, including organizational attempts to mitigate turnover and staff retention efforts; 2) the impact of turnover on practice functioning, including impact on team composition, roles and responsibilities, overall workload, and the ability to provide person-centered care; and 3) practice experiences attempting to recover from these losses, including challenges and successes of attracting and hiring new clinicians, the influence of environmental context, practice setting, and competition from other health systems. Interviews were scheduled for 60 min and conducted on Zoom (33) with two study team members, one conducting the interview and one managing logistics. The interviewer had significant training and experience in qualitative data collection and had no prior significant relationship with participants. Before starting the interviews, the interviewer described their role on the study, the purpose of the study, the focus areas of the interview, and obtained verbal consent from all participants. Audio recordings and auto-generated transcripts were taken of each interview. The interview guide is included as an Supplementary Appendix S1. Transcripts and results have not been shared with participants for additional comment or correction.

The study team concluded data collection after completing 13 interviews and reaching data saturation. Although the parent study focused broadly on clinician turnover within primary care practices located in Virginia HPSAs, APP turnover and hiring emerged as frequent topics during interviews. The central role of APPs in discussions about turnover led our team to conduct a sub-analysis focused specifically on contributors to turnover, impacts of turnover, and retention strategies among NPs and PAs. Across 13 total interviews conducted, 10 contained discussion of APPs and were included in this analysis.

Thematic analysis was conducted by two members of the study team using MAXQDA (34, 35). An initial codebook was developed based on the main topics covered in interviews. The first round of coding was completed across all 13 interviews by both analysts with regular meetings to discuss challenges, discrepancies, and ensure agreement. Coding disagreements were resolved by consensus between coders. After the first level of coding was complete, analysts reviewed the coded data and developed a list of thematic sub-codes focused on APPs. The second round of coding was completed by both analysts across the sub-set of ten interviews that addressed the APP workforce with regular meetings to discuss coding experiences and emergent findings. Following both rounds of coding, the analysis team organized the fully coded data into a results table outlining key themes and related quotes. Narrative memos were developed to describe themes, forming the foundation of the study's results. Throughout data collection and analysis, the study team independently and collaboratively reflected on their potential biases to maximize objectivity.

### 2.3 Study population

We interviewed representatives of primary care practices, including physicians, practice administrators, office managers, and HR managers. We invited 64 practices to participate in the interview and 13 practices (20.3%) accepted. Invitations were stratified across regions and practice size (number of clinicians) with a particular emphasis on practice ownership. Our team's point-of-contact from each practice for the 2022 primary care survey was contacted with information about the study and a request for participation. This individual was responsible for determining who was best suited to discuss the interview topic. For 11/13 practices, this individual completed the interview. For one practice, a clinician referred their HR manager to complete the interview because they felt someone who handles personnel could better speak to the interview topic. For another practice, a practice administrator chose to interview alongside their practice manager, allowing each of their unique perspectives to be shared.

The analysis focuses on experiences with APPs, including both NPs and PAs. Virginia is considered a “restricted practice” state, meaning the state practice and licensure laws require supervision by another healthcare clinician and/or restrict the clinician's ability to conduct one or more element of their practice (36, 37).

## 3 Results

**Figure 1:**
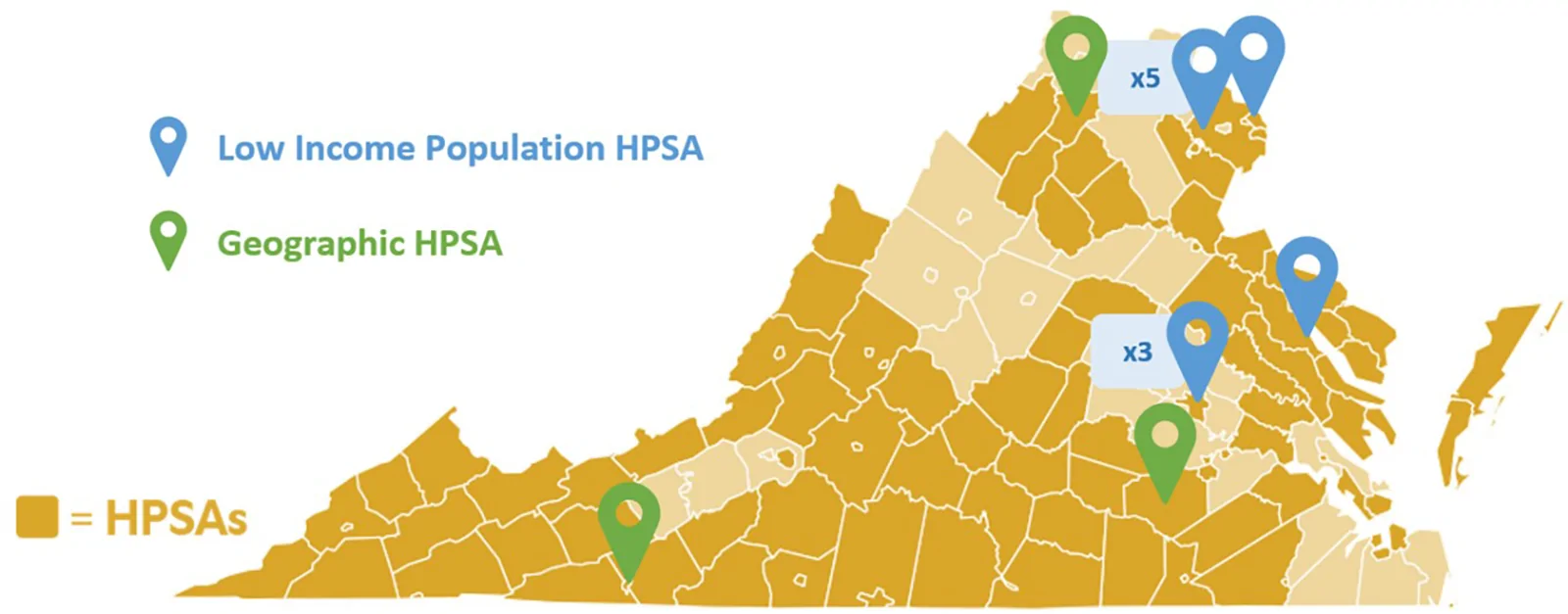
Participating Virginia primary care practices located within Low Income Population HPSAs and Geographic HPSAs.

We interviewed 14 representatives from 13 primary care practices (Table 1) located in Virginia HPSAs (Figure 1). Ten (76.9%) were in Low-Income Population HPSAs and three (23.1%) were in Geographic HPSAs. Nine (69.2%) were adult family medicine or internal medicine clinics, three (23.1%) were pediatric clinics, and one (7.7%) was a women's health clinic. Interviewees were four (28.6%) Family Medicine (FM) Physicians, three (21.4%) Pediatric (Peds) Physicians, two (14.3%) Office Managers, two (14.3%) Practice Administrators, one (7.1%) Human Resources (HR) Manager, one (7.1%) Internal Medicine (IM) Physician, and one (7.1%) OB/GYN Physician.

**Table 1:** Practice and interviewee characteristics of primary care practices.

Practice type	Ownership	Region	Locale (EDGE)^a^	HPSA designation^b^	Interviewee role
Family medicine, residency	Health system	Northwestern Virginia	Rural—Fringe	Geographic	HR manager
Pediatrics	Clinician-owned	Northern Virginia	Suburban—Large	Low Income Population	Pediatric physician
Family medicine	Health system	Central Virginia	City—Midsize	Low Income Population	Family medicine physician
Family medicine	Health system	Central Virginia	City—Midsize	Low Income Population	Family medicine physician
Family medicine	Health system	Central Virginia	Rural—Distant	Low Income Population	Practice administrator
Family medicine	Clinician-owned	Northern Virginia	Suburban—Large	Low Income Population	Practice administrator and office manager
Family medicine	Clinician-owned	Northern Virginia	City—Midsize	Low Income Population	Family medicine physician
Internal medicine	Clinician-owned	Northern Virginia	City—Small	Low Income Population	Office manager
Pediatrics	Clinician-owned	Northern Virginia	Suburban—Large	Low Income Population	Pediatric physician
Family medicine, FQHC	Health system	Southern Virginia	Rural—Distant	Geographic	Family medicine physician
Women's health (primary care)	Clinician-owned	Southwestern Virginia	Rural—Distant	Geographic	OB/GYN physician
Pediatrics	Clinician-owned	Northern Virginia	Suburban—Large	Low Income Population	Pediatric physician
Internal medicine	Health system	Central Virginia	City—Midsize	Low Income Population	Internal medicine physician

a https://nces.ed.gov/programs/edge/Geographic/LocaleBoundaries.

b https://data.hrsa.gov/topics/health-workforce/shortage-areas/hpsa-find.

Prominent themes related to APP hiring, turnover, and retention are outlined below. Findings cover five broad categories with sub-themes: 1) The shift to an APP model: Financial and strategic advantages of APP integration, 2) Benefits of the APP model: APP impact on patient access and provider strain, 3) Contributors to high turnover of APPs: Short tenures, earnings, specialty fields, perceptions of knowledge and skill gaps, and role differences, 4) The impact of APP turnover: Practice functioning, practice operations, and patient care, and 5) Strategies for onboarding and retaining APPs: Intentional recruitment, onboarding, training, and professional development. Most practices reported more experience with NPs than PAs, therefore the majority of the discussion around APPs in the data refers specifically to NPs. The ratio of NPs to PAs in our study reflects the ratio of NPs to PAs across Virginia—there are currently over 13,500 licensed NPs in Virginia as compared to nearly 4,000 licensed PAs in Virginia (38, 39). During interviews practice representatives did not make any meaningful distinction between NPs and PAs when describing experiences with APPs. Though some quotations refer specifically to NPs or PAs, findings are reported across both groups using the umbrella term “APP” to reflect this dynamic.

### 3.1 The shift to an APP model

#### 3.1.1 Financial and strategic advantages of APP integration

The strategic integration of APPs into primary care practices was reportedly driven by the need to mitigate physician shortages, meet community demands, and optimize human resource spending. These collective challenges led some practices to “*come to the realization that we were not going to find physicians who were interested in coming*” and subsequently “*quickly changed our way of thinking to creating a nurse practitioner-based model of care*” (Practice Administrator, Central Virginia). According to participants, the shift to an APP model was facilitated by the larger, more readily available pool of APP applicants, which significantly streamlined the hiring process compared to the often-protracted recruitment of physicians. As one participant explained, “*We open a physician job and we get one applicant a year. We open an APP job, and we get three or four applicants right away. I would be foolish to not consider those alternatives to bring care into the community*” (FM Physician, Central Virginia). Participants noted that APPs are more cost-effective than physicians, allowing organizations to expand their provider base efficiently, as “*an MD is going to be maybe two times the cost, if not more, of an NP… we could safely support another two to three APPs, but where we would maybe only be able to onboard one more MD or DO with that same budget*” (HR Manager, Northwest Virginia). The financial advantage of APPs, they noted, is further solidified by comparable reimbursement rates for NP-provided services from many commercial payers and Medicaid, noting, “*the Medicaids don't cut us 15% for a nurse practitioner*” (Peds Physician, Northern Virginia).

### 3.2 Benefits of the APP model

#### 3.2.1 Positive impact on patient access and provider strain

Participants shared that hiring APPs markedly improved patient access, same-day appointments, hospital follow-ups, and urgent care needs. One HR manager noted that after losing clinicians with large patient panels, “*We onboarded a new PA in August and a new NP in April, so they're kind of helping to offset a little bit of that strain*” (HR Manager, Northwest Virginia). Another physician described a partnership to add an NP faculty member who “*focuses on urgent visits. So that has increased our capacity for those people that need to get in if they're having an urgent problem*” (IM Physician, Central Virginia). Participants felt that APPs alleviated clinician strain and mitigated the impact of clinician loss, thereby ensuring greater continuity of care for established patient panels. Beyond simply managing existing demand, APPs reportedly empower practices to broaden their service offerings and expand access to care. One physician stated that hiring a second NP “*allowed us to expand our primary access pretty significantly*” (FM Physician, Central Virginia), while an OB/GYN credited APPs with allowing the practice to “*take care of a lot of OB situations, do our ultrasounds, non-stress tests, and all that antenatal testing, we do it in my office*” (OB/GYN Physician, Southwest Virginia).

### 3.3 Contributors to high turnover among APPs

#### 3.3.1 Short tenures, higher earnings, and specialty fields

According to participants, the widespread integration of APPs into primary care settings, while strategically advantageous, presents a distinct set of challenges related to clinician retention. Participants indicated that APPs in primary care frequently have short tenures, often lasting only one to two years. As one pediatrician noted, “*The lifespan of a nurse practitioner in our practice was one to two years. Nobody stayed longer than two years.*” (Peds Physician, Northern Virginia). The issue was observed to be particularly pronounced among APPs who were new to the field, some of whom reportedly left primary care to work in a specialty field, often for higher pay and/or preferable work environments. For example, a family medicine physician stated, “*I think this speaks to a bigger issue … that a lot of times nurse practitioners will work in primary care for a few years and then they'll switch to specialty to make more money or be in a more urban location*” (FM Physician, Southern Virginia). Furthermore, remote practices noted a more acute turnover issue among new graduates: “*They just don't really seem to stay, but I think we attract them because that's who we have that will apply to jobs in the middle of nowhere. People with no experience*” (FM Physician, Southern Virginia).

#### 3.3.2 Perceptions of knowledge and skill gaps

Several intrinsic challenges within primary care settings emerged as perceived contributors to APP turnover. The quality of APP training programs was frequently described as variable, with participants explaining that training programs, often delivered online, sometimes provided insufficient clinical experience. For example, one physician remarked that NP programs “*are kind of variable in training. A lot of them are online … their experience in those can be kind of limited. Like, I know some that did like three weeks of pediatrics, and then they're done*” (FM Physician, Southern Virginia). This experience led to differing perspectives on a new APP's capacity for immediate autonomous practice among participants, as some physicians believed APPs were insufficiently trained compared to an MD or a DO. One physician expressed this tension: “*The nurse practitioners want to be able to practice autonomously the day after they graduate from school, even though they've got pretty much no training compared to an MD or a DO*” (FM Physician, Southern Virginia). Most respondents expressed concern regarding critical knowledge and skill gaps among recent graduates.

Relatedly, responses indicated that newly licensed APPs in particular prompted increased oversight from experienced physicians. As one family medicine physician noted, “*They don't know what they don't know, so they don't know to look it up. Like, having to go behind and look at their EKGs that they've ordered*” (FM Physician, Southern Virginia). Complex cases—specifically those involving behavioral health issues, controlled substances, or multiple comorbidities—reportedly caused discomfort for APPs or exceeded their current experience level. Participants also noted that some APPs were “*not feeling prepared to deal with either the amount or the severity of the mental health issues that they were facing*” (Peds Physician, Northern Virginia), and an HR manager reported that some felt “*the cases were either too complicated or beyond the scope of what they felt comfortable with*” (HR Manager, Southwest Virginia). One physician shared their belief about the lack of extensive longitudinal care experience among NPs transitioning from hospital-based roles: “*It's that critical thinking, that longitudinal care of a patient in primary care that I find to be a problem with a lot of the NPs. And for the most part I think that's because most of them come from a hospital-based practice*” (FM Physician, Southern Virginia). Participants indicated that these factors, when combined with demanding workloads and heightened productivity expectations, contributed significantly to increased stress levels among APPs, ultimately impacting their retention in primary care practices. One HR manager recounted a departure where the APP's “*reason for leaving was stress and workload. She did not feel that she could maintain the pace of the other providers and the expectations that we have on the providers for productivity*” (HR Manager, Southwest Virginia).

#### 3.3.3 Role differences, status, and compensation as sources of tension

Tensions arising from clinician role differences within primary care practices emerged as a significant contributor to APP turnover among numerous participants. A portion of participants reported that APPs often experience a perceived demotion in status—for example, when they transition from “*provider*” to “*staff*” upon joining larger health systems—which can lead to feelings of being undervalued. One physician noted, “*When we [health system] took over those practices … they took offense, I think that they were being kind of downgraded in their minds from providers to staff*” (FM Physician, Central Virginia). Participants shared that this sentiment is often exacerbated by a perceived loss of autonomy and an underutilization of their full scope of practice within larger organizational structures. A practice administrator described that a health system “*didn't utilize the nurse practitioners in the way that we use them as full primary care providers with their own panels and everything out here*” (Practice Administrator, Central Virginia). Differences in compensation also played a crucial role, with APPs frequently feeling undervalued due to substantial differences in earnings compared to physicians performing similar services. A pediatrician explained that over time, some APPs “*began to recognize how much money providers were making, the physician providers, doing the same services, and some of the providers left because they found better economic circumstances at other practices*” (Peds Physician, Northern Virginia). Conversely, physician practice owners at times expressed resistance to reducing their own income to equalize APP salaries, highlighting a complex interplay of status, autonomy, and financial considerations that profoundly impact APP retention in primary care. As one physician stated, “*I don't want to be selfish, but I want to be honest … giving up part of your income is not an easy thing to do. We did not at all welcome the thought of reducing our income below the average*” (Peds Physician, Northern Virginia).

### 3.4 Impacts of APP turnover on practice functioning and quality of care

#### 3.4.1 Consequences of APP turnover on patient care quality

Similar to all care shortages, consequences of high professional turnover among APPs on patient care quality were consistently described as detrimental. Staffing shortages and frequent turnover reportedly contributed to reduced appointment availability, particularly for acute visits, with one physician noting that turnover “*definitely puts a squeeze on us as far as being able to have appointments,*” (FM Physician, Southern Virginia) resulting in longer wait times and fragmented care. Participants noted that shortages often forced patients to seek care in urgent care centers or emergency rooms, with practices reporting negative feedback from patients, such as, “*‘Hey, I had a fever and I wasn't able to get into your practice. I had to go to urgent care’*” (HR Manager, Southwest Virginia). This issue was reported to be particularly prominent in rural areas, where patients may already face limited or no affordable care options, leaving “*many of them… with no option*” when a practice loses an APP (Practice Administrator, Central Virginia). The increased workload borne by remaining clinicians due to APP departures reportedly translates into less time per patient and fewer crucial follow-up appointments, leading to a situation where one physician stated, “*I have to spend less time with the patients in the room. Less follow ups*” (FM Physician, Northern Virginia).

Moreover, high turnover was described to erode patient relationships, as patients lost familiar clinicians who possess an in-depth understanding of their medical history, necessitating new care teams to re-establish this critical knowledge base. Participants reported that turnover creates difficulty for patients who “*miss a reassuring voice that they know*” and a clinician who “*knows the patient,*” meaning the new team “*have to get to know the patients all over again*” (IM Physician, Central Virginia). Finally, participants noted that frequent APP turnover places additional financial strain on practices that serve a substantial volume of Medicaid patients, potentially compelling them to shift away from this patient population due to reduced reimbursements and the financial burden of constant recruitment and training.

#### 3.4.2 Operational challenges and financial strain from APP turnover

Participants expressed that professional turnover among APPs in primary care practices also presents significant operational challenges, primarily contributing to substantial re-hiring and onboarding burdens. Participants indicated that new APPs frequently change jobs in pursuit of more favorable economic situations as inequities in pay were commonly reported, creating a perpetual need for training and retraining within practices. This high turnover reportedly impacts not only patient care but also clinician satisfaction, as busy practices struggle with inefficient staffing. Furthermore, participants shared that the supervision of new APPs adds to the workload of already overwhelmed physicians, who are often hesitant to take on this additional responsibility, fearing further reductions in their own efficiency. As one family physician recounted about a colleague, “*One physician was just overwhelmed honestly, and we offered the physician ‘Let's go ahead and get you another nurse practitioner.’ And they said, ‘No, I don't want one because they'll add work to my day. I can't take time to supervise a newer practitioner when I'm already overwhelmed with patients*” (FM Physician, Central Virginia). As evidenced by our data, early departures of APPs due to financial and/or personal incentives may contribute to lost revenue and sediment of wasted training resources within primary care practices.

### 3.5 Strategies for onboarding and retaining APPs

#### 3.5.1 Strategies for effective APP recruitment

Participants emphasized that effective onboarding and retention strategies for APPs in primary care practices are crucial to practice functioning and patient care. Participants identified prior experience, particularly in primary care or with complex and underserved populations, as a strong indicator of an APP's capability, with one physician stating, “*She actually worked at an FQHC in a different state for like 10 years. So she could practice fairly well on her own.*” (FM Physician, Southern Virginia). However, others indicated that a “*blank slate*” mentality, signifying a willingness to be trained and seamlessly integrated into the specific operational model of the practice, was also highly valued. Market dynamics, such as an abundance of APP candidates in metropolitan areas, reportedly affords employers the opportunity to be selective, allowing them to “*cherry-pick*” the most qualified individuals, with one participant emphasizing*, “I have nurse practitioners knocking on my door every day trying to get a job*” (Peds Physician, Northern Virginia). Additionally, for patient populations prone to compassion fatigue, practices reported finding success with experienced individuals already accustomed to working with such populations.

#### 3.5.2 Retention strategies: onboarding, training, and professional growth

To improve APP retention, participants reported that their primary care practices are implementing two main types of strategies: phased onboarding/comprehensive training and professional growth/role validation. Participants emphasized the importance of using gradual onboarding processes that include supportive ramp-up periods, often featuring abbreviated schedules for several months, which allows new APPs to acclimate effectively to new electronic health records and complex workflows. After hiring APPs, practices strived to prevent overwhelm by carefully balancing patient volume with case complexity. To support APPs transitioning into the role of a full provider, practices encouraged new APPs to voice questions and concerns, and often paired new hires with senior clinician mentors.

Other retention strategies referenced by participants focused on APP professional growth and role validation. Key practices included recognizing and utilizing specialized skills over high-volume, general patient loads to prevent burnout and demonstrate respect for advanced training. For instance, a physician noted, “*I have a nurse practitioner who has a PhD and she does mental health. She's not seeing a lot of ear infections and sore throats, nor do I ask her to see more volume*” (Peds Physician, Northern Virginia). Furthermore, empowering APPs with autonomy to pursue specializations within their primary care practice, such as diabetes or sleep clinics, was reported to contribute to job satisfaction and retention. Financial and structural recognition was also cited, with one practice successfully advocating for “*provider-based contract[s]*” with productivity incentives, a status typically reserved for physicians, which was considered “*a plus for us as far as retention*” (FM Physician, Central Virginia). Finally, participants described the importance of professional development opportunities, like participation in institutional committees, in allowing APPs to grow their careers and gain exposure to institutional colleagues and opportunities.

## 4 Discussion

This study highlights current obstacles in the increasingly central role of APPs to mitigate the PCP shortage across the United States, particularly in areas experiencing clinician shortages. We found that primary care practices in Virginia HPSAs are utilizing APPs to expand access, broaden services, and increase financial resources. However, the increasing reliance on APPs is not without challenges, some of which are unique to the APP experience in primary care. Practices reported high turnover among newly hired APPs, which exacerbated practice stress and increased the workload of existing clinicians, contributing to negative impacts on patient care. According to our findings, access to primary care depends on the ability of practices to not only recruit, but also to retain its APP workforce by valuing their full scope of practice and investing in their professional growth.

A review assessing the key factors influencing APP career development within hospital health care teams found that workforce supply, workforce policies and scope of practice regulations, organizational and departmental characteristics, and clinician and patient perceptions and preferences all shape how APP roles are developed and managed (40). These factors span the full workforce lifecycle—recruitment, integration, retention, and career development—a framework we use to interpret our findings on APP tenure and stage-specific needs throughout this discussion. Institutions with extensive experience developing and managing APP roles emphasize the importance of support from senior leadership and physician colleagues, maximizing APP scope of practice, mentorship and professional development for novice APPs, and alignment of clinical skills with patient care needs (41, 42). Our study is consistent with these findings while providing a glimpse into APP hiring, turnover, and retention within Virginia primary care practices in areas that are particularly vulnerable to care shortages.

Our study suggests that Virginia practices located in HPSAs are shifting to APP-driven models of care to capitalize on the rapid growth of APPs in the healthcare provider workforce—now comprising nearly 40% of total U.S. healthcare providers (12). Consistent with prior literature on the financial benefits of APPs, this study's participants noted that the relative ease of recruiting and the reduced cost of hiring APPs, compared to the protracted, often fruitless search for physicians, provides a clear, financially advantageous path for practices to expand their patient base and maintain income levels. This advantage is solidified by comparable reimbursement rates for NP-provided services in Virginia and is a critical motivating factor for rural primary care practices operating on thin margins (30). Though participants considered APP salary levels a motivator for hiring, they also emphasized prominent pay discrepancies between APPs and physicians, and in some cases cited pay as a leading contributor to APP turnover. According to the U.S. Bureau of Labor Statistics, median physician salaries were over $100,000 higher than median NP and PA salaries in 2024 (43). Similarly, some physicians in our study expressed hesitation to reduce their own salaries to improve pay equity between APPs and PCPs. In order to support APP longevity within primary care practices, policy leaders and practice leadership should prioritize stronger salary compensation for APPs and equitable productivity incentives.

Literature confirms that employing APPs is not only financially advantageous for many primary care practices, it also positively impacts patient experience and outcomes (17, 19). Numerous participants in our study recognized the key role APPs played in improving care capacity and quality within their practices, echoing national data. A 2022 national survey of over five thousand physicians reported that most physicians working with NPs and/or PAs thought they benefited their clinical practice, and notably, physicians with higher incomes working in medical school settings were more likely to view PA impact on patient volume, care quality, time use, and workload as positive (18). Another study focused on APP service utilization in academic medical centers (AMCs) concluded that APPs have been integrated into most services offered at AMCs, and NPs and PAs are positively rated on the value they bring to their centers. In describing the contribution of APPs, some physicians expressed the sentiment that PAs and NPs do not duplicate physician efforts, but rather compliment them by managing more common cases and sharing insights from diverse prior work experiences (44, 45).

Despite the increasing number of practicing APPs over the last decade, training outcomes suggest a decline in the number of NPs pursuing rural primary care (46), mirroring trends seen within the primary care physician workforce (8). This is concerning, as primary care physician shortages are projected to reach an estimated 20,200 to 40,400 primary care physicians by 2036 (13), with a 1.9% mean annual loss of practicing family physicians in rural areas (29). Echoing reports that approximately 1 in 5 NPs switch jobs annually, our study highlights rural practice challenges with high professional turnover among APPs (20), suggesting that those entering rural practice may not represent a long term or sustainable resource without intervention. While unclear if this is true for only APPs, it appears that rural areas may experience greater clinician insufficiencies than their non-rural counterparts. Though programs like the Graduate Nurse Education Demonstration Project seek to incentivize and support NP training in rural primary care (46), our findings suggest that APPs may gain experience working in rural areas before transitioning to more urban locations and/or higher paying specialist fields (46). The literature on NP pay shows prominent salary differences by geographic area and specialty, emphasizing a need for a systemic approach to APP pay equity (20). Beyond compensation, however, sustained rural engagement likely also depends on lifecycle-long investment—from locally rooted training pipelines to ongoing professional development—rather than pay reform alone (47).

Participants in our study described how APP turnover can negatively impact patient care and practice functioning on several fronts. As seen within the physician and nursing labor force, frequent turnover exacerbates reductions in appointment availability and erodes the critical patient-clinician relationships that are foundational to effective primary care (24). This loss of familiar clinicians disrupts continuity of care, requiring new teams to re-establish a patient's history and leaving patients who “*miss a reassuring voice that they know*” (25, 48). Operationally, high turnover creates a perpetual need for recruitment and training, wasting resources and adding to the workload of already overwhelmed physicians, who may be hesitant to take on the additional responsibility of supervising newer APPs (49, 50). A global systematic review reported that primary care physician turnover creates unnecessary financial and operational strain among practices in socio-economically deprived areas (51). In the U.S., primary care physician turnover alone has been estimated to generate $979 million in annual excess health care costs, more than a quarter of which (27%) is attributable specifically to burnout-related departures (49). According to participants in our study, the financial impacts of turnover were particularly prominent within clinician owned practices that must contend with lower revenue and more intense competition from those owned by health systems (47).

Multiple factors intrinsic to primary care settings were found to contribute to high turnover, some of which are unique to APPs. Concerns were raised about the variability of APP training, particularly perceived insufficiencies in clinical experience, which is an identified limitation in the literature (52). We found that this led to stress for newer APPs managing complex cases involving behavioral health, controlled substances, or multiple comorbidities and a perceived need for increased physician oversight, which may be inadequate at busy practices. As documented elsewhere, training programs may not always adequately prepare APPs to independently manage complex patients in practice (53). Consequently, new NP graduates often report feeling unequipped to work in practice settings involving patients with high complexity and acuity, pressures exacerbated by demanding workloads and high productivity expectations (54, 55). Notable, however, feelings of competence and confidence are similar between newly practicing APPs and physicians (56).

Relatedly, our findings highlight the tension between a common desire among APPs for more autonomous practice and these perceived gaps in training. This is a complex issue, as greater professional autonomy in the workplace is a critical factor in APP job satisfaction and burnout in the primary care setting (57, 58), yet fully autonomous practice has been shown to lead to turnover among novice NPs (23). It is therefore crucial for practices hiring APPs to implement formal, phased training programs with supportive ramp-up periods to bridge clinical experience gaps, particularly for new graduates transitioning from hospital-based roles (59–61). Practices must actively strive to prevent new APPs from feeling overwhelmed by carefully balancing patient volume with case complexity, and providing mentorship and support throughout the training and onboarding process. Participants reported positive experiences hiring APPs with training and experience relevant to their practice's patient populations, but noted that prior experience in a position is not a unique indicator of success in a role. This is consistent with primary care-specific evidence linking intentional recruitment, skill-mix training, and workplace wellness practices to sustained APP engagement (62).

Differences in role status, such as discrepancies in compensation compared to physicians for similar services, and perceived demotion among APPs from “*provider*” to “*staff*” when practices join larger health systems, were found to further foster feelings of being undervalued. Physician respondents at times portrayed negative perceptions of APP training and competency in primary care, which influenced willingness to hire and train new APPs. These findings align with previous work showing that APPs and physicians had significantly different perspectives on APP competence in caring for patients independently, time management skills, and physician support in the transition to practice (56). This signifies a need for improved awareness of APP competencies among physicians, greater APP role validation, and review of compensation models to establish APPs as autonomous clinicians within care teams and promote retention. Practices in our study that successfully implemented “*provider-based contracts*” with productivity incentives found that status recognition improved morale and retention, providing a form of structural recognition that validates the APP role and helps address the financial and status-based tensions that drive turnover. To further mitigate professional dissatisfaction among APPs, practices may also offer opportunities to pursue specializations within the practice (e.g., diabetes or sleep clinics) or leadership roles on institutional committees, rather than demanding high-volume, general patient loads, thereby addressing the career advancement concerns reported elsewhere in the literature (63–65). More broadly, these strategies reflect calls in the workforce literature to move beyond standardized, high-volume caseloads and toward flexible, non-traditional role structures that accommodate mid-level providers' evolving career needs (66).

## 5 Limitations

Our study is subject to several limitations. First, the findings rely exclusively on the perspectives of physicians and practice administrators. This is because the parent study selected practices based on self-reported turnover among all clinicians, not specifically among APPs. While research has increasingly called for investigating employer perceptions of APP practice readiness (32), relying solely on these perspectives here introduces a potential one-sided bias, as the study does not include the direct experiences, motivations, or viewpoints of the APPs themselves regarding professional, practice, or personal factors contributing to turnover. Secondly, the sample was geographically restricted to Virginia HPSAs, which may not be generalizable to other states. Future research should explore how geographic and sociodemographic differences influence the rates of and contributors to APP turnover.

## 6 Conclusion

While integrating APPs into HPSA practices is a necessary strategy to address the primary care access crisis, limited PCP availability combined with reported systemic turnover of APPs threatens practice functioning and patient care. To successfully retain APPs, practices must move beyond simply hiring them as a stopgap measure, and instead implement comprehensive retention strategies, including clinician autonomy, equitable compensation, and professional development opportunities. These changes require a cultural shift towards viewing APPs as autonomous, long-term partners in care delivery. Increased understanding of the knowledge and skills of APPs across specialties and training programs may shift PCP perceptions.

## Data Availability

The datasets presented in this article are not readily available because Deidentified transcripts may be made available upon request of authors with appropriate human subjects protections in place. Requests to access the datasets should be directed to Marie Shoen, marie.shoen@vcuhealth.org.
