# Supplementary material for: The role of Advanced Practice Providers in improving primary care capacity: practice perspectives on challenges and opportunities
Source: Front Med (Lausanne). 2026 Sep 3;13:1885926. doi: 10.3389/fmed.2026.1885926 (PMC13581626; doi:10.3389/fmed.2026.1885926)
Supplement: Supplementary file 1 [file Supplementaryfile1.docx]

Supplementary Material

# 1 Appendices

## 1.1 Interview Guide

We are conducting interviews with primary care practices located in federally designated healthcare professional shortage areas to understand their experiences with clinician and staff turnover and its impact on their ability to care for patients.

Between 2018 and 2022, practices reporting having recently lost a clinician rose from 13% to 42%, with some clinics operating down 3 to 5 nurses at all times. During follow-up interviews, practices underscored how these losses negatively impacted the morale and functioning of the practice, contributing to emotional and financial stresses and risked undermining their ability to provide high-quality, team-based care.

Through interviews with practices like yours we hope to understand the long-term challenges and needs faced by practices experiencing high turnover, and its impact on practices’ ability to care for underserved communities.

1. What clinician losses has your practice experienced and what factors do you think have contributed to their departure?
   a. Probe for:
      i. Relative impact of organizational, health system, and personal factors
      ii. Awareness of being in HPSA and what that means to them
      iii. Organizational attempts to mitigate turnover and retain staff
2. What has been the impact of clinician turnover on how your practice functions?
   a. Impact on team-based care
      i. Probe for description of changes to team composition, roles and responsibilities of each member (pre-visit planning, messaging, closing care gaps, follow-up outside of visits), and overall workload
   b. Impact on patient care and relationship with patients
      i. Probe for ability to provide person-centered care, including time spent with patients, communication, trust
   c. Impact on organizational capacity, including ability to make practice improvements, changes, etc.
3. What efforts has your practice tried to recoup from clinician loss?
   a. Factors shaping ability to attract and hire new clinicians
      i. Probe for environmental context, practice setting, structure of position, competition from other practices or health systems

## 1.2 Results Table

| **Results Table** | |
| --- | --- |
| *Theme* | *Quotes* |
| **The Shift to an APP Model** | |
| **Shift Towards NP-Based Models of Care:** Due to the difficulty in hiring physicians, some practices have proactively shifted their models of care to rely more heavily on NPs to meet community needs. | - We opened a physician job and we get one applicant a year. We open an APP job, and we get three or four applicants right away. I would be foolish to not consider those alternatives to bring care into the community. - Family medicine physician, Central VA |
| **Ease of Recruiting NPs:** There is a much larger and more readily available pool of NP applicants, making their recruitment significantly easier and quicker than for physicians. | - It took us a couple of years actually to come to the realization that we were not going to find physicians who were interested in coming. So we quickly changed our way of thinking to creating a nurse practitioner based model of care out here with a few providers as the anchor points. And now I think we have the largest amount of nurse practitioner primary care in the system. So that's where we are today. It's worked very very well. - Practice administrator, Central VA - *[Why more of an emphasis on hiring APPs at the rural practice site?]* If I'm being honest, because they can't get anybody else. Would they like to hire MDs out there? Absolutely. - Family medicine physician, Central VA - Hiring nurse practitioners is much, much, much easier than hiring physicians. To provide context, we've had two full-time physician positions posted since the summer of 2021. We have interviewed a total of three candidates for both positions in the last three years. - Family medicine physician, Central VA |
| **Payer Reimbursement for NPs:** Some commercial payers and Medicaid do not significantly reduce reimbursement rates for services provided by NPs, making them a financially viable option for practices. | - I have nurse practitioners knocking on my door every day trying to get a job. And the reason they are is that when you're looking at the commercial [payers] now, the Medicaids don't cut us 15% for a nurse practitioner. - Pediatric physician, Northern VA |
| **Financial Advantages of Hiring NPs:** From a budgetary perspective, NPs are considerably more cost-effective than physicians, allowing organizations to hire more providers for the same budget. | - From a financial standpoint, an MD is going to be maybe two times the cost, if not more, of an APP. So from a budgetary standpoint, we could safely support another two to three APPs, but where we would maybe only be able to onboard one more MD or DO with that same budget. - Human resources manager, Northwestern VA |
| **Protecting Practice Income:** Hiring NPs can be a strategy for practices to protect or maintain their income levels, especially when physician recruitment is challenging and costly. | - We chose to hire NPs to protect our own income. - Pediatric physician, Northern VA |
| **Benefits of the APP Model** | |
| **Increased Patient Access:** Hiring NPs improves patient access, especially for same-day appointments, hospital follow-ups, and urgent care needs | - We have pretty consistently struggled to get patients in the door. Even our own patients who want same-day sick appointments or who need a hospital follow-up. So when we have lost providers who had huge established patient panels, it's really tough. We onboarded a new PA in August and a new NP in April, so they're kind of helping to offset a little bit of that strain. - Human resources manager, Northwestern VA - Once we started hiring folks, we started seeing so many good applicants that we just kept working that model. And I think it's worked out pretty well. They've allowed us to expand our primary access pretty significantly. - Family medicine physician, Central VA |
| **Offsetting Provider Strain/Loss:** NPs help to alleviate the burden on existing staff and can compensate for the loss of other providers, maintaining continuity of care for established patient panels. | - I hired a second nurse practitioner, and they're a tremendous help. I do not know how I would survive if I didn't have them. They see a lot of patients with me supervising them. We take care of a lot of OB situations, do our ultrasounds, non-stress tests, and all that antenatal testing, we do it in my office so they (patients) get a quick answer. - OB/GYN, Southwestern VA |
| **Expansion of Services:** NPs enable practices to expand their service offerings and provide more comprehensive care. | - We did actually have a partnership with the school of nursing, so that we did add an NP faculty member who wanted to work in the complex care clinic, and she focuses on urgent visits. So that has increased our capacity for those people that need to get in if they're having an urgent problem. - Internal medicine physician, Central VA |
| **Contributors to High Turnover among APPs** | |
| *High Turnover of NPs in Primary Care* | |
| **Short Tenures:** NPs often have a short lifespan in practices of one to two years, primarily due to economic reasons (desire for higher pay) or family situations (spouse relocating for a job). | - The lifespan of a nurse practitioner in our practice was one to two years. Nobody stayed longer than two years. And most of the reason was the economics, the nurse practitioner wanting more money that we didn't have. Or family situation, the husband took another job somewhere. - Pediatric physician, Northern VA |
| **Turnover More Acute Among Inexperienced NPs:** Practices in more remote areas tend to attract applicants with little to no experience. | - They just don't really seem to stay, but I think we attract them because that's who we have that will apply to jobs in the middle of nowhere. People with no experience. - Family medicine physician, Southern VA |
| **Using Primary Care as a Stepping Stone:** Nurse practitioners often leave primary care for specialty fields due to higher earning potential or a desire for urban locations. | - I think this speaks to a bigger issue that I know from friends and colleagues working around the state is that a lot of times nurse practitioners will work in primary care for a few years and then they'll switch to specialty to make more money or be in a more urban location. - Family medicine physician, Southern VA |
| *Perceived Limitations in NP Training Programs* | |
| **Variable Quality of NP Training:** NP training programs are often seen as inconsistent in quality, frequently offered online, and providing insufficient clinical experience. | - Nurse practitioners are RNs that then go on to a nurse practitioner program, which are kind of variable in training. A lot of them are online. They are like two years. They include clinical rotations in various specialties, but their experience in those can be kind of limited. Like, I know some that did like three weeks of pediatrics, and then they're done. They graduate. They can practice medicine. - Family medicine physician, Southern VA |
| **Differing Views on NP Capacity for Autonomous Practice:** NPs may desire to practice autonomously immediately after graduation, while some physicians believe NPs are insufficiently trained compared to an MD or a DO. | - The nurse practitioners want to be able to practice autonomously the day after they graduate from school, even though they've got pretty much no training compared to an MD or a DO. During the pandemic the requirements for autonomous practice decreased, kind of inching ever closer towards no requirement. They can just practice right after school… NP schools and NP societies sell themselves as a cure to the primary care shortage. But the reality is not that. - Family medicine physician, Southern VA |
| *Perceived Knowledge and Skill Gaps in Key Areas of Primary Care* | |
| **Knowledge Gaps in New Practitioners:** Newer NPs may lack the experience to identify what they don't know, requiring experienced physicians to double-check their work. | - They don't know what they don't know, so they don't know to look it up. Like, having to go behind and look at their EKGs that they've ordered. - Family medicine physician, Southern VA |
| **Complexity of Cases:** NPs, especially newer ones, may be uncomfortable with or lack experience in managing complex cases, particularly those involving behavioral health, controlled substances, or multiple comorbidities. | - The cost of the NP is lower. However, nurse practitioners don't do what I do. When I walk into a room, I'm seeing a kid that is followed at [hospital] with 15q syndrome, and has seizure disorder, significant developmental delays, is non-verbal, can't get into a dentist, is not eating, has to see a nutritionist … every patient that I walk into is extremely complex now. Because you know who’s seeing the sore throats, the ear pain, the abdominal pain, the constipation. - Pediatric physician, Northern VA |
| **Lack of Longitudinal Care Experience:** Many NPs may lack experience in longitudinal patient care in primary care settings, potentially due to coming from hospital-based settings. | - It's that critical thinking, that longitudinal care of a patient in primary care that I find to be a problem with a lot of the NPs. And for the most part I think that's because most of them come from a hospital-based practice. They've not had that experience in a family medicine or internal medicine residency, where you're monitoring that patient over a long period of time. - Family medicine physician, Southern VA |
| *Productivity Expectations* | |
| **Workload and Stress:** NPs may feel unable to keep up with the demanding pace and high expectations put on other providers for productivity, leading to stress. | - One of the things we've heard from practices is them hiring NPs and some not feeling prepared to deal with either the amount or the severity of the mental health issues that they were facing. - Pediatric physician, Northern VA - They felt that the cases were either too complicated or beyond the scope of what they felt comfortable with. It could have been like a hospital follow-up that was really complicated. Someone with you know, just a tremendous amount of comorbidities. - Human resources manager, Southwestern VA - Her reason for leaving was stress and workload. She did not feel that she could maintain the pace of the other providers and the expectations that we have on the providers for productivity. - Human resources manager, Southwestern VA |
| *Tensions Around Provider Role Differences* | |
| **Perception of Status and Role:** NPs may feel downgraded when transitioning from “provider” to “staff” when joining health systems. | - When we [health system] took over those practices, we had a standard model where all nurse practitioners were hired on as staff the way that you would hire a nurse or a medical assistant as a staff member. But at the practice [we took over] they were [originally] hired on as providers, so they took offense, I think that they were being kind of downgraded in their minds from providers to staff. - Family medicine physician, Central VA |
| **Loss of Autonomy and Scope of Practice:** NPs may be underutilized in large health system settings. | - We had nurse practitioners in every family medicine practice, and the only ones that were offered contracts, when we switched over into [health system], were the MDs. And that sent the nurse practitioners into a complete tizzy because [health system] didn't utilize the nurse practitioners in the way that we use them as full primary care providers with their own panels and everything out here. - Practice administrator, Central VA |
| **NP Compensation Discrepancy:** NPs may feel undervalued by significant differences in earnings for performing the same services as physicians. | - We paid the nurse practitioners about half of what we paid pediatricians and at least initially they were satisfied with that, cause that was kind of what the community standard was. I think some over time began to recognize how much money providers were making, the physician providers, doing the same services, and some of the providers left because they found better economic circumstances at other practices. - Pediatric physician, Northern VA |
| **Physician Resistance to Income Reduction:** Physician practice owners may be unwilling to reduce their own income to equalize NP salaries. | - I don't want to be selfish, but I want to be honest. When you're older, well experienced, and you have a lifestyle based on a predictable economic level, giving up part of your income is not an easy thing to do. We gave bonuses, when we had extra money, to the nurse practitioners as opposed to giving it to ourselves. But we did not at all welcome the thought of reducing our income below the average. - Pediatric physician, Northern VA |
| **Impact of APP Turnover on Practice Functioning and Quality of Care** | |
| *Compromises to Quality of Patient Care* | |
| **Appointment Availability:** Staffing shortages and turnover lead to reduced appointment availability, longer wait times, and patients ending up in urgent care or emergency rooms. | - That's where we've seen all of the turnover is in our mid-level provider positions. Especially in the wintertime, when we're seeing a lot more sick visits, acute visits, that sort of thing, it definitely puts a squeeze on us as far as being able to have appointments. - Family medicine physician, Southern VA |
| **Limited Access in Rural Areas:** In some cases, patients are left with no affordable options for care, especially in rural areas or when outside practices don't accept insurance. | - We do have a handful [of patients] who have gone out and sought care elsewhere. Of course we're getting some of those negative reviews and feedback like, ‘Hey, I had a fever and I wasn't able to get into your practice. I had to go to urgent care.’ - Human resources manager, Southwestern VA |
| **Increased Workload for Remaining Clinicians:** If NPs leave and are not immediately replaced, the remaining clinicians have to manage a larger patient panel, leading to less time per patient and fewer follow-ups. | - The patients were either left a) seeing a locum provider which really ticked them off. Or, b) sending them to one of the new outside practices, which they all went to concierge medicine and were not accepting insurance. And so our particular patient population couldn't necessarily afford that. So many of them were left with no option. Everything else is an hour away at least. - Practice administrator, Central VA - I have to spend less time with the patients in the room. Less follow ups. More difficulty for the patients to schedule an appointment. So a lot of those people either have to look somewhere else to find care. Or they just have to wait or end up in emergency rooms. - Family medicine physician, Northern VA |
| **Undermines Patient Relationships:** High turnover among NPs challenges practice relationships with patients, as patients miss familiar faces and providers who know their medical history and needs, requiring the new team to re-establish this knowledge. | - Our patients didn't like the change. They got to know someone. And then it's like, well, they went to another position. They miss a reassuring voice that they know so that's hard for the patients. And then someone that, more importantly, knows the patient. And it takes you a while to know that, ‘OK, this patient, although she has asthma, and she's calling every day, and she's short of breath, she's talking in full sentences and she's just calling for some reassurance.’ But that takes time to get to know. And when we have turnover, the nurse practitioner and the nursing team have to get to know the patients all over again. - Internal medicine physician, Central VA |
| **Financial Pressures on Practices Serving Medicaid Patients:** Frequent NP turnover puts added financial strain on practices that serve high volumes of Medicaid patients forcing them to consider shifting away from this patient population. | - My practice is one of the larger practices in my area that sees Medicaid patients. So what's going to happen is eventually I have to stop and stay with commercial because of how the current market around me is, how the commercial payers are reducing my reimbursements for nurse practitioners. Now I can't pay more to get more clinicians because I see a Medicaid population and they don't understand. - Pediatric physician, Northern VA |
| *Re-Hiring and Onboarding Burden* | |
| **Training Burden:** Young NPs may frequently change jobs for higher pay, leading to constant training and retraining. | - Many, many of the young people are jumping from job to job to get a better economic situation. So they will take a job, stay until they go home at night, and find a better job on the internet and just are moving from job to job to job. It's very difficult to maintain those people. And that has its influence on patient care and on provider satisfaction if you end up having to train and retrain and/or have staff that are not very efficient in a busy practice. - Pediatric physician, Northern VA |
| **NP Supervision and Physician Burden:** Already overwhelmed physicians are hesitant to supervise new NPs, fearing increased workload and reduced efficiency. | - One physician was just overwhelmed honestly, and we offered the physician ‘Let's go ahead and get you another nurse practitioner.’ And they said, ‘No, I don't want one because they'll add work to my day. I can't take time to supervise a newer practitioner when I'm already overwhelmed with patients.’ - Family medicine physician, Central VA |
| **Inefficient Onboarding and Early Departure:** The onboarding process for new NPs can take several months, which can result in lost revenue and wasted training time and resources when NPs depart early. | - It takes about 3 to 4 months to really onboard someone. One NP realized it wasn't going to be a good fit, and she was with me for about 4 months. So in the time that it took to get her acclimated, she was like ‘this ain't for me’ and she left. - Internal medicine physician, Central VA |
| **Strategies for Onboarding and Retaining APPs** | |
| *Targeted Hiring Based on Experience and Fit* | |
| **Quality Assurance in Hiring:** Prior experience, particularly in primary care settings or working with complex or underserved populations, is seen as an indicator of NP capability and quality. | - She actually worked at an FQHC in a different state for like 10 years. So she could practice fairly well on her own. - Family medicine physician, Southern VA - [Ours] is a patient population that puts people at risk for some compassion fatigue, and it is a hard patient population to deal with. So we have had more luck with either new grads or few folks coming to us from the community that are used to this population. - Internal medicine physician, Central VA |
| **Flexibility and Willingness:** Prioritize candidates who demonstrate a "blank slate" mentality, indicating a readiness to be trained and integrated into the specific operational model of the practice. | - The key right now has been the flexibility and that willingness to learn new things and want to do something different. Kind of like a blank slate that you can educate them in your model. - Internal medicine physician, Central VA |
| **Market Dynamics:** The abundance of NP candidates in more metropolitan areas allows employers to "cherry pick" the most qualified individuals. | - What we found is the NP market here in Northern Virginia is flooded. I get NP resumes every single day. So now I can cherry pick the very best. - Pediatric physician, Northern VA |
| *Phased Onboarding and Training* | |
| **Gradual Onboarding and Support:** Practices emphasize a slow, supportive ramp-up for new NPs, allowing ample time to acclimate to new EMRs and workflows before increasing patient load. This often includes abbreviated schedules for several months. | - We've been able to be flexible enough with them to allow them the time to really get their feet under them to allow them a slower ramp up time when it comes to seeing patients. We've been very flexible. We've handheld some of them when they've needed it, instead of just saying come on you gotta do it…And so as long as these people stay, I think it'll be a really good thing. - Practice administrator, Central VA |
| **Importance of Speaking Up:** New hires are encouraged to voice questions and concerns, rather than trying to navigate challenges alone. | - We've let them lead the pace. We give them an hour block per appointment for the first couple of weeks to make sure they're comfortable with the EMR and the office workflow. But then we kind of leave it up to them like, ‘Hey, how are you guys feeling? Are you ready to ramp up? Are you comfortable where you are? What do you need?’ - Human resources manager, Southwestern VA |
| **Focus on Retention and Avoiding Overwhelm:** There's a conscious effort to prevent new NPs from feeling overwhelmed, balancing patient volume with complexity to support their transition and encourage long-term retention. | - We onboarded a new PA in August. And we just onboarded a new NP in April. We're being very very cautious because we don't want to overwhelm them. We don't want them to get in the same situation where they're feeling like this is too complicated and too much too soon. We're really trying to balance keeping them full by giving them the simple same day stuff, and keep the more complicated stuff on our experienced providers, and try to support them as much as we can so that we can retain them. - Human resources manager, Southwestern VA - When they join they usually have an abbreviated schedule for longer than probably the business folks would want. But that's what they need. Just because there's a lot of information that they need to review. So giving them time, giving them not weeks, but months to get acclimated is what's needed. And being able to make sure that we have selected or hired a person that's willing to speak up when they have some questions and not feel like they have to go it alone. - Internal medicine physician, Central VA |
| **Mentorship and Peer Support:** New NPs are frequently paired with senior clinicians for mentorship, ensuring continuous support and opportunities for consultation, particularly for complex cases or sick children. | - For the first several months to even a year they are paired up one-on-one, or even one-to-two, with our senior providers and they are given allotted time each week to have these mentorship type of meetings. But the way that our teams are set up is that you are always typically paired with a senior provider. So the mid levels or the NPs would always have the support. - Human resources manager, Southwestern VA |
| **Addressing Under-coding:** NPs may under-code visits due to fear of audits, necessitating training to ensure proper billing and compensation for services rendered. | - Every nurse practitioner that I've talked to has told me they are taught in all of these nurse practitioner schools to under-code for fear of being audited, or something. So that's usually like one of the first things I have to do with them is get them to stop under-coding their visits so that we can actually get paid for the work we do. - Family medicine physician, Southern VA |
| *Leadership and Professional Growth* | |
| **Valuing Specialized Skills Over Volume:** Not forcing highly specialized NPs to see high volumes of general cases demonstrates respect for their advanced training and prevents burnout. | - I have a nurse practitioner who has a PhD and she does mental health. She's not seeing a lot of ear infections and sore throats, and nor do I ask her to see more volume. - Pediatric physician, Northern VA |
| **Empowering Autonomy and Specialization:** Allowing NPs to pursue their passions and specialize in areas like diabetes, sleep, or mental health can contribute to job satisfaction and retention. | - Would you want to do a diabetes clinic? A sleep clinic? A weight clinic?’ … We try to give our providers the opportunity to do what they're passionate about. - Human resources manager, Southwestern VA |
| **Provider-Based Contracts and Incentive Structures:** Treating NPs as "providers" with productivity incentives, similar to physicians, acknowledges their contribution and value. | - The provider-based contract for the APPs was something that [practice] advocated for [when joining the health system]. Those are the only APPs in the health system who are considered providers and have a productivity incentive for the work that they're doing. So that is a plus for us as far as retention. - Family medicine physician, Central VA |
| **Opportunities for Professional Development and Leadership:** Providing avenues for career growth through committee participation and exposure to other institutional colleagues may foster engagement and long-term commitment. | - The NP took a role within the Academic Medical Center on a committee so that they had exposure to other members … but also so that we could help them grow and identify needs to help them with their career growth. - Internal medicine physician, Central VA |
